# Physicochemical Changes and Antioxidant Metabolism of *Actinidia arguta* Fruit (Kiwiberry) Under Various Cold-Storage Conditions

**DOI:** 10.3390/molecules30183742

**Published:** 2025-09-15

**Authors:** Barbara Łata, Rafał Wołosiak, Ewa Majewska, Beata Drużyńska, Małgorzata Piecyk, Katarzyna Najman, Anna Sadowska, Piotr Latocha

**Affiliations:** 1 Department of Plant Protection, Institute of Horticultural Sciences, Warsaw University of Life Sciences, 02-776 Warsaw, Poland; barbara_lata@sggw.edu.pl; 2 Department of Food Technology and Assessment, Institute of Food Science, Warsaw University of Life Sciences, 02-776 Warsaw, Poland; rafal_wolosiak@sggw.edu.pl (R.W.); ewa_majewska1@sggw.edu.pl (E.M.); beata_druzynska@sggw.edu.pl (B.D.); malgorzata_piecyk@sggw.edu.pl (M.P.); 3 Department of Functional and Organic Food, Institute of Human Nutrition Sciences, Warsaw University of Life Sciences, 02-776 Warsaw, Poland; katarzyna_najman@sggw.edu.pl (K.N.); anna_sadowska@sggw.edu.pl (A.S.); 4 Department of Environmental Protection and Dendrology, Institute of Horticultural Sciences, Warsaw University of Life Sciences, 02-776 Warsaw, Poland

**Keywords:** modified atmosphere storage, controlled atmosphere storage, ozonation, ascorbate, glutathione, phenolics, antioxidant activity

## Abstract

*Actinidia arguta* (kiwiberry) is a fruit with significant health benefits, and research continues to identify factors that enhance its storability while maintaining quality. Special attention is given to antioxidant metabolism and total antioxidant activity. In this study, four cold-storage conditions were tested: normal air, normal air with ozone, modified atmosphere, and controlled atmosphere. In each case, the fruit was either pre-cooled before storage or not. The aim was to identify conditions most favorable to preserving internal and external fruit quality over time. Taking into account most of the basic fruit physicochemical traits tested, it can be assumed that for up to 30 days of storage, each storage method can be useful to store *A. arguta* fruit. After this period, the fruit stored in a controlled and then modified atmosphere retained the highest and acceptable firmness. Changes in antioxidant content are more complex and depend on the type of compound, storage time, and fruit post-harvest treatment. During the 50-day storage period, marked fluctuations in ascorbate, glutathione, and L-cysteine levels were observed at 10-day intervals. Phenolic content increased initially (after 10 days) and then stabilized. Among the methods used, ozonation led to a relative stabilization or increase in antioxidant content. This method, like the cooling procedure, requires further detailed research to determine its suitability for the species/variety being tested. Changes in antiradical activity were reaction-mechanism-dependent. The activity based on single electron transfer consistently decreased, while that based on hydrogen atom transfer was more stable overall. Contrary to this, the pro-oxidative Fe(II) chelating agent appeared during storage. The health-promoting properties of stored fruit may fluctuate due to antioxidant involvement in adaptation to storage conditions and uneven ripening, which remains a challenge both at harvest and during storage. Any of the three alternatives to cold storage in CA (NA, NA+O, MA) proved beneficial in short-term storage. However, MA has proven to be a similarly effective long-term storage method to CA in terms of the physicochemical quality of *A. arguta* fruit.

## 1. Introduction

In food production, many conditions must be met to obtain a high-quality product. In the case of plant-based food, such as fruit or vegetables, the spectrum of factors guaranteeing their quality is very wide [1]. Starting with factors that act during the plant growth period, then after harvest, during transport and finally during storage [2,3,4]. One of the key features of these products is storage potential, whereby in the case of storage time, both the nature of the fruit, e.g., its structure, and its quality at the harvest time, which is a consequence of agroclimatic conditions, and then the preparation of the fruit for storage and the storage conditions themselves, have an impact [5,6,7].

*Actinidia arguta* Miq. is a relatively new fruit in terms of commercial cultivation, but with relatively well-understood growth factors, including benefits and risks, with a wide range of red- and green-skinned varieties differing in fruit size, flavor and bioactive compound contents [8,9]. As a result, it is a fruit of interest to both growers and consumers [10].

Many scientific works have been devoted to the qualitative–quantitative analysis of the fruit chemical composition, which determines its healthcare function (pro-health values). Superfruit, as the *A. arguta* fruit is called, is characterized by a high content of vitamins (A, C, E), thiol compounds (glutathione), carotenoids, lutein, inositol, and phenolic compounds [9,11]. A total of 539 compounds, with several health-promoting properties, have been isolated and identified from the *A. arguta* fruit [12,13]. The high content of biologically active compounds is related to high total antioxidant activity, and the *A. arguta* fruit is qualified as a valuable source of antioxidants for a healthy and balanced diet [9,12]. The fruit is also rich in minerals [8,14].

Among the many advantages of *A. arguta*—such as low susceptibility to diseases and pests during the growing season, resistance to low winter temperatures, and fruits with high biological value—uneven ripening remains a significant challenge. This uneven ripening complicates the determination of the optimal harvest date for storage, leading to variability in fruit quality during cold storage [15,16,17]. Uneven ripening, rapid softening after harvesting, and the associated loss of firmness pose a challenge not only in terms of when to harvest the *A. arguta* fruit but also in terms of how to proceed after harvesting to extend its storage life [18]. Therefore, various methods are being sought to improve its storability potential. One of the objectives of this study was to determine whether cooling the fruit prior to subsequent packaging at a higher temperature affects its storage potential. The effect of cooling fruit before storage in the case of *A. arguta* has, to our knowledge, been explored to a limited extent. Considerable research has focused on optimizing storage technologies to extend storability while preserving quality. Many storage solutions exist, but they must be tailored to the species or even specific varieties, often combining multiple approaches [19,20].

At the current stage of supply of *A. arguta* fruits on the market, they are stored relatively shortly after harvesting, but as the supply increases, longer storage will become necessary. Key factors affecting fruit storage and shelf life include temperature— the primary driver of postharvest metabolism [4,17,19,21]—humidity, which influences transpiration rate, and storage atmosphere composition [22,23,24]. Increasing CO_2_ and lowering O_2_ levels slows respiration, prolonging shelf life. Many chemical and physical methods were tested in *A. arguta* preservation and storage [25,26,27,28,29,30,31]. Among them, ozone (O_3_) is emerging as an effective physical preservation method for maintaining postharvest fruit quality [29,30]. Controlling ethylene production and respiration remains a primary goal during storage. Packaging technologies like Xtend^®^ bags, which regulate gas composition and humidity, offer further benefits. The last-mentioned have scarcely been studied in the case of the *A. arguta* fruit. From a biological standpoint, selecting late-maturing, durable varieties is another strategy [32].

In the fruit quality term, in addition to maintaining firmness, appearance, and overall palatability, it is also essential to preserve biologically active compounds. Enzymatic and non-enzymatic antioxidants are involved in the fruit’s health value and may also act as signaling molecules during fruit ripening and aging. Each method of postharvest treatment and storage specifically affects the antioxidant metabolism [17,24,27,33,34,35]. The content of antioxidants, such as phenolic acids, flavonoids, ascorbic acid, and glutathione, as well as the total antioxidant capacity can be maintained or increased over a period of time in response to carefully selected and properly applied factors/conditions before and during storage. However, the effectiveness varies depending on the specific compound and storage method used [24]. Among the mentioned compounds, the most difficult to maintain over a longer period of time is the vitamin C content since water-soluble vitamin C is highly sensitive to external factors; it typically degrades rapidly during storage [21,26].

Cold-stored *A. arguta* fruits (1 ± 0.5 °C) retained stable phenolic levels and antioxidant capacity over a six-week storage period, although their vitamin C content decreased [21]. Eo et al. [19] suggest that the quality characteristics and antioxidant activity of kiwifruit are significantly influenced by low-temperature storage conditions; however, this should be further investigated for specific kiwifruit varieties.

The combination of low oxygen and high carbon dioxide levels, as applied in technologies like controlled atmosphere (CA), ultra-low oxygen (ULO), and dynamic controlled atmosphere (DCA), has proven effective in preserving bioactive compound concentrations. However, the effectiveness in the case of *A. arguta* varies depending on the specific compound and storage method used [24]. Notably, an atmosphere with 5% CO_2_ and 1.5% O_2_ (CA) helped maintain high ascorbic acid levels for up to 12 weeks.

1-MCP (1-methylcyclopropene) can be used to delay the softening of the *A. arguta* fruit and extend its storage and shelf life [27,28]. However, 1-MCP can also reduce the edibility of some fruits and cause ripening disorders, which has led to a decline in consumer acceptance of 1-MCP-treated fruit. Recently, ozone has been used as an effective and environmentally friendly method of physical fruit quality preservation after harvest [29]. It has been found that ozone activates antioxidant enzymes and the ascorbate–glutathione cycle, preventing the increase in reactive oxygen species (ROS) and further consequences of oxidative stress, such as membrane lipid peroxidation. Similarly to kiwifruit [29], ozonated *A. arguta* fruits exhibited lower ROS levels due to their higher content of polyphenolic and Vitamin C and higher activity of enzymes involved in ROS scavenging, such as superoxide dismutase and catalase. This improved fruit quality and their antioxidant activity [30]. However, the authors argue that to fully understand the mechanisms occurring in fruit after ozonation, more detailed research is necessary, including molecular level, as well as further storage experiments. Ozone concentration, ozonation time, and cultivar response may all play a role.

Given that *A. arguta* is a seasonal fruit and a relatively new market product, advancing research and storage technologies is critical to expand cultivation areas, extend the supply period of high-quality products, and enhance the fruit’s economic value. Even though the topic of storing the *A. arguta* fruit has been taken up by researchers, the search for new, more efficient and cheaper methods seems to be still an open question.

The aim of the study was therefore to evaluate the antioxidant status, antioxidant/antiradical activity and other fruit characteristics in relation to various, more or less researched in the case of *A. arguta*, storage techniques at 10-day intervals, using the Weiki variety, proven in commercial cultivation. Common cold storage with or without ozonation, modified atmosphere storage using Xtend^®^ bags in a standard cold store and controlled atmosphere storage, being relatively inexpensive methods, were simultaneously compared with respect to fruit quality and the potential storage time of each method. Ozonation and Xtend^®^ bags were tested as new or less recognized solutions in *A. arguta* storage. Additionally, the effect of postharvest fruit cooling on fruit storability and quality was under consideration.

## 2. Results and Discussion

### 2.1. Basic Physicochemical Parameters

One of the main challenges in post-harvest storage of *A. arguta* fruit is its uneven ripening on the vine. Fruits from commercial plantations are typically harvested at the harvest maturity stage while still firm. The harvest date is determined based on the average soluble solid content (SSC) of a relatively small sample of fruits. As a result, the harvested batch often contains fruits with quite varied SSC levels (ripeness stages). Consequently, these fruits continue to ripen at different rates during storage, leading to uneven softening. The softer fruits become more susceptible to infections, and in larger packages, they can serve as a source of pathogens that compromise the entire batch, disqualifying it.

A potential solution to this issue could be the development of non-invasive fruit sorting technologies for A. arguta based on SSC levels, such as near-infrared (NIR) spectroscopy, which has already been developed for some other fruit crops [36,37].

Across all tested storage combinations, no signs of spoilage were observed for up to 50 days. Under controlled atmosphere (CA) storage, fruits remained disease-free for an additional 10 days, whereas mold symptoms appeared in some fruits stored under other conditions. Therefore, the results were reported for up to five storage terms to include all experimental combinations. Moreover, in the normal air plus continuous ozone treatment at 0.3 ppm (NA+O), minor skin damage was observed, likely due to the toxic effect of ozone on the delicate fruit skin. Other studies have shown benefits from using ozone even at higher concentrations, but for shorter periods [30].

**Figure 1:**
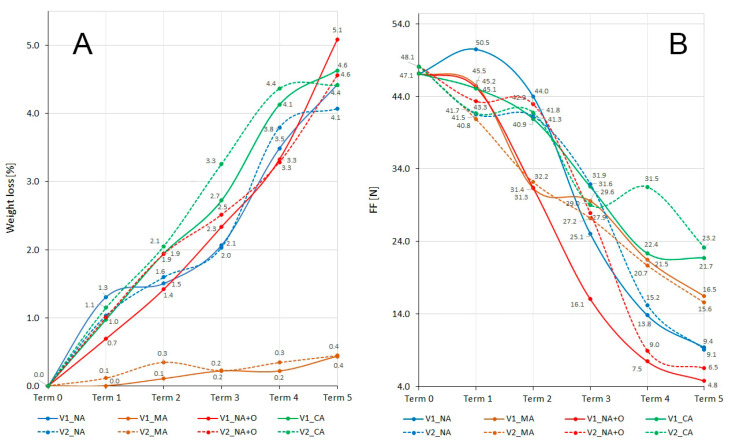
Changes in physical properties of *Actinidia arguta* fruit at 10-day intervals depending on storage conditions and fruit cooling before storage: (**A**) fruit weight loss; (**B**) fruit firmness (FF). Designations: V1—postharvest variant with storage at 8 °C for 14 h (pre-cooling), V2—postharvest variant without pre-cooling after harvest, NA—normal air, MA—modified atmosphere, NA+O—normal air + ozone, CA—controlled atmosphere. The numbers in the graph represent the real values measured for the particular compounds and measurement points.

Rapid weight loss (WL) and skin wrinkling are common storage problems reported for *A. arguta* [16,25,38]. In our study, fruits stored in a modified atmosphere (MA) exhibited the lowest average WL throughout the storage period in both postharvest treatments (V1—postharvest variant with storage at 8 °C for 14 h (pre-cooling), V2—postharvest variant without pre-cooling after harvest), with losses up to only 0.4% after 50 days (Figure 1A, Table 1). Similar findings were reported for *Actinidia chinensis* var. *deliciosa* (A.Chev) A.Chev. (kiwifruit) by Han et al. [39]. In contrast, fruits stored under other conditions, despite 90–95% relative humidity, experienced WL several times higher, ranging from 4.1% in normal air (NA) to 5.1% in normal air with ozone (NA+O) after 50 days. However, unlike previous studies [25], no skin wrinkling was observed in any treatment, even at the end of the storage period. During the first 10 days, fruits stored in normal air with continuous 0.3 ppm ozone (NA+O) lost less weight than those in other conditions, but this trend reversed thereafter. Overall, postharvest treatment and storage combination had no significant impact on WL over the entire storage period.

Similarly to WL, a decrease in firmness (FF) is a typical phenomenon observed after harvesting soft and climacteric fruits, including the *A. arguta* fruit. The postharvest ripening process in *A. arguta* is inextricably linked to a progressive reduction in firmness until the fruit becomes noticeably soft at consumption maturity. Maintaining low temperatures, reducing oxygen concentration, and increasing carbon dioxide levels in the storage atmosphere all have beneficial effects on extending the shelf life of various fruit types [40]. A similar effect has been reported for ozone treatment, whether applied once before storage or continuously during the entire storage period [41]. According to the authors, ozone not only inhibits the synthesis of autocatalytic ethylene but may also improve the overall health status of stored fruit. In our study, the most rapid average firmness loss across all storage periods was observed in the fruit stored in normal air with added ozone (Figure 1B, Table 1), while the slowest decline was recorded in the fruit stored under controlled atmosphere (CA) conditions. However, as shown in Figure 1B, during the first 20 days of storage, the fruit stored immediately after harvest for 14 h at 8 °C retained higher firmness. This trend reversed over time. On average, throughout the entire storage period, the fruit that was transferred directly to cold storage after harvest—but subjected to a gradual, stepwise temperature reduction (i.e., without fluctuations)—exhibited higher overall firmness.

**Figure 2:**
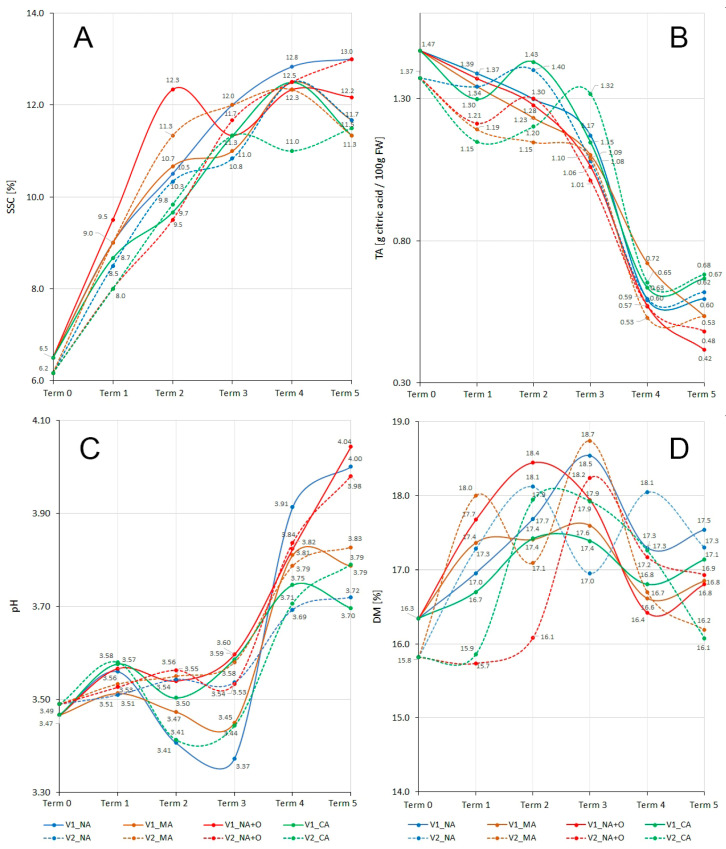
Changes in basic physicochemical properties of *Actinidia arguta* fruit at 10-day intervals depending on storage conditions and fruit cooling before storage: (**A**) soluble solid content (SSC); (**B**) titratable acidity (TA); (**C**) pH; (**D**) dry matter (DM). Designations: V1—postharvest variant with storage at 8 °C for 14 h (pre-cooling), V2—postharvest variant without pre-cooling after harvest, NA—normal air, MA—modified atmosphere, NA+O—normal air + ozone, CA—controlled atmosphere. The numbers in the graph represent the real values measured for the particular compounds and measurement points.

The basic quality parameters determining the degree of fruit ripeness are SSC and titratable acidity (TA). Their mutual ratio in fruits affects the perception of fruit sweetness [42]. In our study, with storage time, the SSC increased from the initial value of 6.2 and 6.5% to the level of 11.3-13% and TA decreased (from 1.37 to 1.47 to 0.42–0.68 g citric acid/100g fresh weight, FW), which are typical patterns of change during the ripening of all fruits, including *A. arguta* [43] (Figure 2A,B and Table 2).

The direction of these changes was consistent across all storage combinations and both postharvest treatment variants; however, the rate of change differed. On average, over the entire storage period, the fruit placed in cold storage on the day of harvest and stored under controlled atmosphere (CA) conditions maintained lower soluble solids content (SSCs) and higher acidity for a longer time (i.e., ripened more slowly). In the case of acidity, however, the differences were less pronounced, as similar levels were also maintained in fruit stored under normal air (NA) and modified atmosphere (MA) conditions (Table 2).

The pH changed relatively little during storage. The *A. arguta* fruit is a low-pH fruit, with initial values averaging 3.47 and 3.49 for V1 and V2, respectively, immediately after harvest. During storage, its pH values initially decreased slightly, then increased, and eventually reached 3.70–4.04, depending on its storage conditions (Figure 2C, Table 2). On average, over the entire storage period, postharvest treatment had no significant effect on pH. Among the storage combinations, only the fruit stored under normal air with added ozone exhibited significantly higher pH values compared with the other conditions (Table 2).

Dry matter content (DM) is a key factor linked to fruit WL. However, it largely depends on the fruit’s maturity at harvest and how quickly it ripens during storage. In our study, DM content varied over time and differed among treatments (Figure 2D, Table 2). During the first 20 days of storage, all tested storage combinations showed an increase in DM, followed by a decrease, and then another rise toward the end of storage in most cases. Overall, the fruit from the V1 variant had a significantly higher DM content (17.2%) compared with V2 (16.9%). Part of this difference may be due to the slightly higher initial DM in pre-cooled fruit (V1: 16.3%) versus non-pre-cooled fruit (V2: 15.8%) at Term 0. However, the main reason probably relates to different levels of fruit maturity at harvest.

The analysis of basic physicochemical parameters and their changes during storage confirmed previous findings regarding uneven ripening on the vines [44]. As a result, *A. arguta* showed considerable variability in both firmness and internal quality—factors that influence postharvest behavior. Although certain changes in the fruit occur regardless of postharvest treatment or storage conditions, it was evident that placing the fruit directly into cold storage after harvest and storing under controlled atmosphere (CA) conditions effectively slowed fruit softening. This method allowed for extended storage while maintaining good quality. Although modified atmosphere (MA) conditions reduced fruit WL compared to other storage options, they had no significant effect on the other basic quality parameters. In other studies, edible coatings applied to fresh produce proved to be a viable alternative to modified atmosphere packaging, minimizing quality degradation and WL by modifying and regulating the internal atmosphere of individual fruit [45]. In contrast, ozone treatment had no significant effect on extending storage life or delaying ripening. Furthermore, it showed signs of toxicity, causing micro-damage to the fruit’s delicate skin. Further research with different ozone concentrations may be necessary to verify and clarify these findings.

### 2.2. Fruit Chemical Composition

#### 2.2.1. Ascorbate

Ascorbate (vitamin C) is an essential compound in both general and stress-related plant and animal physiology [46]. Its significance and functions have been extensively described in numerous scientific studies [46,47,48,49]. Ascorbate plays a crucial role in the antioxidant defense system, which is activated under extreme conditions associated with various abiotic stresses (such as high or low temperature, light intensity, radiation, ozone, and others), as well as biotic stress factors. Consequently, interest in the levels of this antioxidant arises not only from its direct link to fruit quality, but also from its role in enhancing tolerance to oxidative stress—a common physiological response to all types of stress [20,50]. From a nutritional perspective, the greatest challenge regarding this compound is its stability, as it is a hydrophilic antioxidant highly sensitive to degradation when exposed to pro-degradative factors [49].

**Figure 3:**
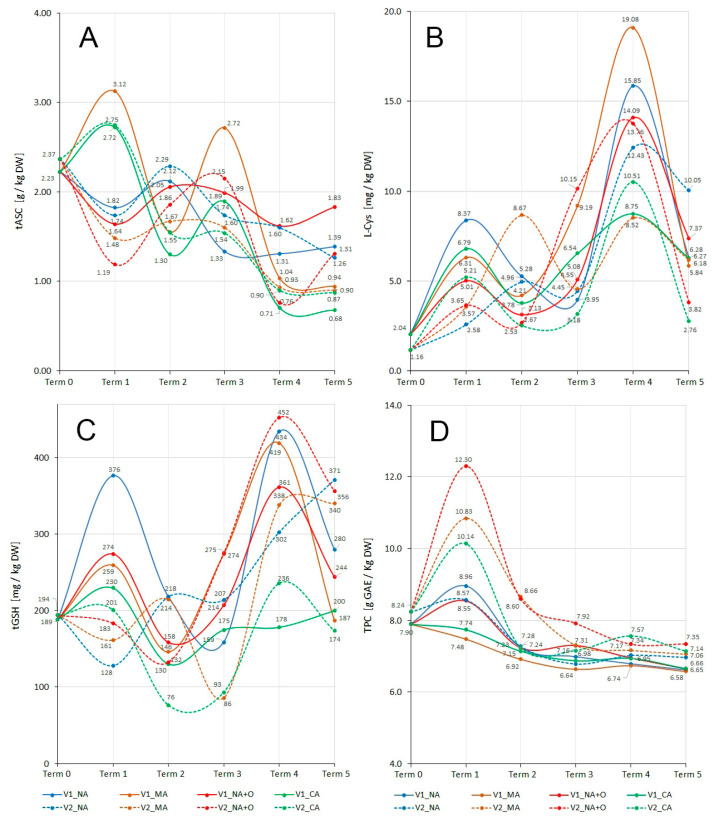
Changes in the chemical composition of the *Actinidia arguta* fruit at 10-day intervals depending on storage conditions and fruit cooling before storage: (**A**) total ascorbate (tASC); (**B**) L-cysteine (L-CYS); (**C**) glutathione (tGSH); and (**D**) total phenolic content (TPC). Designations: V1—postharvest variant with storage at 8 °C for 14 h (pre-cooling), V2—postharvest variant without pre-cooling after harvest, NA—normal air, MA—modified atmosphere, NA+O—normal air + ozone, CA—controlled atmosphere. The numbers in the graph represent the real values measured for the particular compounds and measurement points.

Statistical analysis did not show any significant effect of fruit cooling before storage (as the first main effect) or the storage conditions (as the second main effect) on the total ascorbate content. The time of storage in relation to ascorbate content was highly significant (Table 3). The mean values of this compound, depending on storage variant (V1 vs. V2) and/or storage conditions (NA, MA, NA+O, and CA), are presented in Table 4. However, as can be seen in Figure 3A, on the one hand, we are dealing with a high dynamic of ascorbate changes during fruit storage; on the other hand, clear differences were revealed between selected storage conditions in the picture of these variations. These changes were indeed influenced by storage time. To summarize the statistical analysis, the interactions between the tested main factors were stronger than the main effects, except for storage time (Table 3).

The difference in the average ascorbate content between the pre-cooled and uncooled fruit was small, approximately 6% (Figure 3A). However, during storage, clear differences emerged between the two variants and storage conditions. Pre-storage cooling resulted in smaller decreases in the ascorbate content of the fruits stored under two conditions (V1_NA and V1_NA+O) after the first 10 days compared with the non-cooled fruit. This difference was particularly significant in the NA+O treatment, where the ascorbate decrease in V1_NA+O was 28% relative to the starting point, whereas in V2_NA+O it reached 99%. Similar results were observed by Krupa et al. [51] in studies on the Weiki variety stored in cold conditions, where a significant vitamin C decrease occurred after one week, followed by slight fluctuations up to day 42. In those studies, the fruits were not pre-cooled, while in the present study, pre-cooling had no significant overall impact on the ascorbate content (Figure 3A).

A general downward trend with minor fluctuations in vitamin C content during cold storage has also been reported in late-maturing *A. arguta* resources [32] and other studies [21]. In our research, the greatest ascorbate decrease under cold-storage conditions (V1_NA) occurred after 30 days. Regarding ozone treatment, recent studies suggest a potential effect on the antioxidant cycle and fruit redox state in kiwifruit and kiwiberry, particularly related to fruit aging [29,30]. Ozone-treated kiwifruits stored in cold conditions exhibited slight fluctuations in vitamin C content, similar to our observations, with significantly higher vitamin C levels in ozonated fruits compared to untreated controls [29]. In our experiment, the *A. arguta* fruit stored with NA+O showed higher ascorbate content than non-ozonated fruit (NA) during the later storage stages (Term 3-Term 5), but only in the pre-cooled variant. Conversely, fruits from the V2_NA+O chamber (non-cooled with ozone) showed large fluctuations in ascorbate throughout storage (Figure 3A).

Piechowiak et al. [30] reported improved *A. arguta* quality—reflected by lower reactive oxygen species and higher levels of low-molecular-weight antioxidants, including ascorbate—after ozonation at varying concentrations and times. This indicates that ozonation is a promising preservation method, although the optimization of parameters such as exposure time and ozone concentration is necessary. Our results also highlight that pre-storage fruit preparation plays a significant role in maintaining ascorbate levels during storage.

In the other two storage combinations, the effects of cooling and storage method on ascorbate content showed a different pattern during the first storage period. The fruits stored in controlled atmosphere (CA) exhibited, in Term 1, an increase in ascorbate compared with harvest values, regardless of pre-cooling, with increases of approximately 22% in the cooled fruit and 18% in the uncooled fruit. This rise may be explained by the fruit’s acclimatization to the altered atmosphere, characterized by reduced oxygen and elevated carbon dioxide levels compared with normal air [52].

In contrast, pre-cooling combined with modified atmosphere (MA) storage led to different outcomes: the pre-cooled fruits (V1_MA) showed about a 40% increase in ascorbate, whereas the uncooled fruits (V2_MA) experienced a 45% decrease after 10 days of storage (Term 1). During subsequent storage, ascorbate levels fluctuated, likely reflecting ongoing physiological changes in the fruit after harvest and during early storage stages. The greatest fluctuations were observed in the V1_MA and V1_CA treatments, while V1_NA+O showed relatively stable ascorbate content throughout storage.

Other observations have been made with storage technologies involving oxygen and carbon dioxide control, such as dynamic controlled atmosphere (DCA), ultra-low oxygen (ULO), and CA, where relatively minor changes in vitamin C content occurred during *A. arguta* storage [24]. Specifically, atmospheres with higher CO_2_ concentrations (around 5% in CA) were most effective at maintaining high ascorbic acid levels even after 12 weeks.

Other research demonstrated that shock cooling of the *A. arguta* fruit immediately after harvest (3–4 °C within 2 h) reduced quality degradation and helped retain more L-ascorbic acid during CA storage and shelf life [7]. In the present study, regardless of fruit preparation (V1 vs. V2), a gradual decline in ascorbate was observed under both CA and MA conditions, with large fluctuations between sampling dates compared with other storage methods.

Similarly, in studies on *Actinidia kolomikta* Maxim., ascorbic acid content decreased during ripening regardless of the storage atmosphere [22]. These authors identified the best storage conditions for maintaining the highest ascorbic acid levels as chambers with 1.5% O_2_ and 3% CO_2_.

#### 2.2.2. Thiol Compounds: L-Cysteine (L-CYS) and Total Glutathione (tGSH)

L-cysteine (L-CYS) is a precursor of glutathione (GSH), and its fluctuations are closely linked to GSH metabolism. Both ascorbate and glutathione play crucial roles in maintaining the redox balance within fruit cells through the Asada-Halliwell cycle, which directly impacts internal fruit quality and antioxidant potential [29]. Since fruits often experience oxidative stress during ripening and postharvest aging—leading to increased production of reactive oxygen species—the levels of compounds protecting against oxidative damage are essential. The accumulation of ascorbate (AsA) and glutathione, and the maintenance of redox balance, are therefore vital for preserving postharvest fruit quality [53,54].

All tested factors—fruit cooling, storage condition, and storage time—significantly influenced L-CYS content, as did most interaction effects, except for the interaction between the fruit preparation variant and storage combination (Table 3). Overall, pre-cooling of the fruit before storage (V1) led to a significant increase in L-CYS content, although differences between the cooled and uncooled fruits within the same storage combination were modest (Table 4). The fruits stored under normal air (NA) and modified atmosphere (MA) exhibited significantly higher L-CYS levels on average compared with those stored under controlled atmosphere (CA) conditions (Table 4).

At harvest, L-CYS content was significantly higher—by about 1.75 times—in the cooled fruits compared with the uncooled ones (Figure 3B). During storage, L-CYS levels fluctuated dynamically, with alternating increases and decreases. Throughout the entire storage period, L-CYS concentrations were often much higher than levels measured immediately after harvest. Regardless of pre-storage treatment (V1 vs. V2), L-CYS content increased at the first evaluation time point (Term 1) relative to harvest (Term 0). A second, even greater increase was observed at Term 4 across all storage combinations. The smallest fluctuations in L-CYS levels occurred between Terms 2 and 3 (Figure 3B).

Regarding total glutathione content (tGSH), storage method and duration were significant factors, but fruit pre-cooling had no statistically significant effect when averaged across treatments (Table 4). The fruits stored under CA conditions had significantly lower tGSH contents compared with other storage environments. The effects of cooling and storage conditions on tGSH were also dependent on the timing of measurements, showing significant interactions between variant and time, as well as combination and time (Table 3).

At harvest, tGSH levels were similar between cooled and uncooled fruit, differing by only about 3% (Figure 3C). In the pre-cooled fruits (V1), tGSH content increased after 10 days of storage, with the magnitude of this increase varying by storage condition: the largest increase was seen in V1_NA (+94%), while the smallest was in V1_CA (+19%). In contrast, the uncooled fruits (V2) showed a slight tGSH increase only in V2_CA (+6%), with all other V2 combinations showing decreases ranging from −3% (V2_NA+O) to −48% (V2_NA).

At Term 2, tGSH levels generally decreased compared with Term 1, except in the V2_NA and V2_MA combinations, which showed significant increases. Between Terms 2 and 3, the tGSH concentrations remained relatively stable, exhibiting the least fluctuation over the storage period. By Term 4, most combinations demonstrated the greatest tGSH increases compared with harvest, except V1_CA, which showed no increase.

Overall, glutathione changes followed a pattern similar to that of its precursor L-cysteine, with alternating increases and decreases. Comparable fluctuations in glutathione content have been reported elsewhere [29], where ozone treatment enhanced glutathione levels in kiwifruit. In the current experiment, however, ozonation did not significantly affect tGSH levels in cold-stored fruits (NA+O vs. NA). Unfortunately, no studies on the content of thiol compounds in kiwifruit in terms of storage have been found.

In studies on other fruits, such as raspberries, ozone treatment increased activities of glutathione reductase and glutathione peroxidase, which helped reduce glutathione loss and improve hydrogen peroxide scavenging [55]. Glutathione’s role in responding to environmental stresses is increasingly exploited in postharvest preservation; for example, exogenous GSH application can alleviate chilling injury by activating the AsA–GSH cycle and boosting antioxidant capacity during the low-temperature storage of mango and bell pepper [53,56].

Similarly, investigations into modified atmosphere treatments in pomegranate peels revealed a rapid initial glutathione increase during storage, followed by a decline [52]. A comparable initial glutathione increase was also observed in our study.

#### 2.2.3. Phenolics

The antioxidant potential of the *Actinidia arguta* fruits, apart from vitamin C, is primarily determined by phenolic compounds [11,12,13]. Like other biologically active substances, phenolics contribute not only to the biological value of the fruit but also play a key role in maintaining postharvest quality during storage. Similarly to the function of the ascorbate–glutathione redox system, phenolics are capable of directly reacting with reactive oxygen species (ROS), the accumulation of which is a major factor in the degradation of fruit quality during storage.

Statistical analysis of our results showed that all tested factors and their interactions significantly affected the total phenolic content (TPC) (Table 3). Notably, the fruits that were not cooled prior to storage (V2) exhibited significantly higher concentrations of phenolic compounds across all storage conditions (Table 4). Among the tested combinations, the fruits stored under NA+O conditions demonstrated the highest TPC on average, while the lowest levels were observed in the fruits stored under NA conditions, followed by CA (Table 4). It is important to emphasize that these differences partly reflect the fruit’s physiological response to storage conditions and postharvest treatment (V1 vs. V2), particularly evident after the first 10 days of storage (Figure 3D). The most pronounced increase in TPC was observed in the ozonated uncooled fruits followed by the uncooled MA, CA, and NA stored fruits.

This stimulatory effect of ozonation on low-molecular-weight antioxidants has also been reported by other authors [29,30,55]. In kiwiberries, a clear relationship has been demonstrated between ozonation and polyphenol oxidase (PPO) activity. The authors showed that both the dose and duration of ozonation significantly affected PPO activity—specifically, longer exposure times were associated with reduced enzyme activity [30,55].

The TPC in the fruits immediately after harvest was very similar regardless of whether they were cooled or not, with only a 4% difference between V1 and V2 at Term 0 (Figure 3D). The greatest changes in TPC occurred during the first ten days of storage and depended on storage conditions (Figure 3D). The highest increase in phenolics compared with the postharvest state was recorded in the V2 variant under NA+O (49.3%), MA (31.4%), and CA (49.0%) conditions. In contrast, in the V2_NA combination, phenolic levels changed only slightly between Term 0 and Term 1, with an increase of about 4%. In the V1 variant, the changes in TPC were minor, showing a slight increase in NA and NA+O combinations (8.2–8.5%) and a slight decrease in CA and MA combinations (2.1–5.6%) (Figure 3D). Between Term 1 and Term 2, the TPC decreased close to the initial level (Term 0), with the greatest declines seen in fruit from combinations that exhibited the largest increases during the first term. From Term 2 to Term 3, the TPC stabilized, followed by minor fluctuations until the end of storage. Aside from initial differences observed at the beginning of storage among the tested combinations (Figure 3D), phenolic levels were similar from Term 3 onward, regardless of storage conditions. At the end of the storage period (Term 5), the TPC ranged from 6.58 g GAE/kg DW (V1_MA) to 7.35 g GAE/kg DW, which corresponds to an average decrease of approximately 16% compared with Term 0.

Our results indicate that a high antioxidant content can be expected within the first ten days of fruit storage, which is the result of the fruit’s adaptation strategy to new environmental conditions, in which antioxidants play a significant role. Over the next 20 days, antioxidant content may gradually decline and stabilize at a certain level. After 30–40 days of storage, an increase in antioxidant concentration may occur due to oxidative processes related to, among other things, fruit aging, but this can also lead to unacceptable softening, loss of firmness, and mold development. This effect can be influenced by pre-cooling, but cooling time and temperature require more extensive testing to reveal more positive or negative effects on fruit quality. Nevertheless, a certain trend was observed: the gradually cooled fruits were characterized either by an increase in their content of bioactive compounds (thiols, total ascorbate) or by their stabilization (phenolics). Phenolic compounds were the most stable in terms of content in the *A. arguta* fruit. In this case, the absence of pre-cooling led to an increase in their content at the beginning of storage. Conversely, the pre-cooled fruit showed only minor changes in the total phenolic content (TPC) throughout the storage period. Therefore, the level of these compounds is determined by many factors.

The results of other studies suggest that changes in antioxidants such as phenolics in kiwiberries induced by cold stress are cultivar-dependent, with certain varieties potentially consuming their antioxidants to mitigate damage caused by low temperatures [19,51]. Therefore, the amount of these compounds during storage may be influenced by their endogenous, genetically determined levels. However, the total flavonoid and TPC, regardless of cultivar, remained relatively stable or even increased during cold storage compared with other tested compounds. These findings are consistent with other studies on the effect of storage conditions on phenolic content in fruits [21,26,32].

### 2.3. In Vitro Antioxidant/Antiradical Activities

In this study, we examined antioxidant properties using methods based on different mechanisms. The ABTS and CUPRAC assays primarily (ABTS) or exclusively (CUPRAC) measure potential antiradical activity through the single electron transfer (SET) mechanism. In this process, the antioxidant donates an electron to a free radical, neutralizing it. The antioxidant itself becomes oxidized, but the product is typically more stable than the original radical. In contrast, the crocin bleaching assay (CBA) assesses antiradical activity solely via the hydrogen atom transfer (HAT) mechanism, where the antioxidant donates a complete hydrogen atom—a proton plus an electron—to the free radical. This converts the radical into a stable molecule, while the antioxidant becomes a less reactive radical [57]. The chelation ability assay involves antioxidants binding to pro-oxidative transition metal ions, in this case Fe(II), present in the kiwiberry extract. Testing antiradical activity using various methods is a common research practice and allows for the full antioxidant potential of the raw material to be identified.

**Figure 4:**
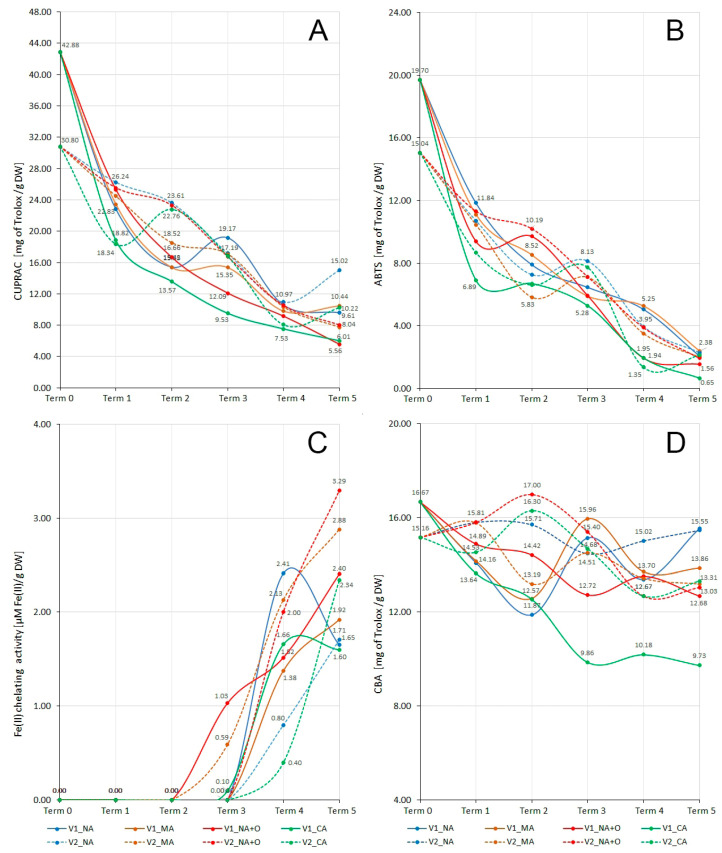
Changes in antioxidant/antiradical activity of the *A. arguta* fruit (kiwiberry) at 10-day intervals depending on storage conditions and fruit cooling before storage: (**A**) CUPRAC assay; (**B**) ABTS assay; (**C**) Fe(II) chelating activity; (**D**) crocin bleaching assay (CBA). Designations: V1—postharvest variant with storage at 8 °C for 14 h (pre-cooling), V2—postharvest variant without pre-cooling after harvest, NA—normal air, MA—modified atmosphere, NA+O normal air + ozone, CA—controlled atmosphere. The numbers in the graph represent the real values measured for the particular compounds and measurement points.

Different storage conditions cause biochemical and degradation changes in kiwiberries, leading to qualitative and quantitative shifts in bioactive compounds responsible for antioxidant properties, and consequently, variations in the fruits’ antioxidant activity. Figure 4A shows the results obtained using the CUPRAC method, which measures reduction capacity at neutral pH. A general decrease in electron-donating activity was observed in all tested kiwiberry samples during storage. Despite a particularly large initial decrease in activity in the cooled fruits, pre-cooling after harvest and its absence did not have a statistically significant effect (Table 3) on the activity measured by this method (in subsequent storage, the fruits from both groups behaved similarly). Storage in a controlled atmosphere (CA) for about 50 days resulted in significantly lower activity compared with storage in refrigerated conditions under an unmodified atmosphere (NA). In the other conditions, the differences were not statistically significant (Table 5).

Considering the antiradical activity measured by the ABTS method (Figure 4B), a decrease in activity was observed during storage, which aligns with the similar antioxidant mechanism reflected in the CUPRAC results. However, some differences emerged, likely due to the mixed electron transfer and hydrogen atom transfer mechanisms involved in the ABTS assay. Unlike CUPRAC, the variants with pre-cooling after harvest showed higher average antiradical activity than those without pre-cooling (Table 5). Consistent with the CUPRAC findings, storage under controlled atmosphere (CA) significantly reduced antiradical activity compared with all other storage conditions. The iron ion chelation ability was initially undetectable but appeared after the second and third storage terms (Figure 4C). This ability increased over time in most variants. Exceptions were observed in the fruits cooled immediately after harvest and then stored under CA conditions, where the increase was inhibited after 40 days, and in the fruits stored in refrigerated normal air (NA), where the chelation ability decreased at the end of storage, reaching in those cases the lowest final values among all variants.

Figure 4D and Table 5 show changes in antiradical activity measured by the crocin bleaching assay (CBA), which relies solely on the hydrogen atom transfer (HAT) mechanism. Minor fluctuations in antiradical activity occurred during storage. The fruits stored under refrigerated conditions with normal air (NA) retained final activity levels similar to the initial values, regardless of pre-cooling. Other samples experienced a decline in activity, but this decrease was less marked compared with the CUPRAC and ABTS results. Jeong et al. [21] reported similar findings for hardy kiwifruits during cold storage, noting minimal fluctuations in antiradical activity when measured by ORAC (HAT mechanism) compared with the ABTS and DPPH methods. Pre-cooling caused in our study only a slight, yet statistically significant, decrease in activity compared with non-cooled samples. The largest reduction in antiradical activity (42%) occurred in the pre-cooled fruits stored under CA, a change that was statistically significant compared with other storage methods (Table 5).

Reports from other researchers indicate that antiradical activity is higher in less ripe fruits [58], which aligns with the findings presented in this study. Similarly, other studies have observed a decrease or slight decrease in antiradical activity during storage [59]. However, specific changes over shorter storage periods are difficult to predict, as they depend on storage conditions and the initial fruit composition at harvest. For instance, Krupa et al. [51] noted a slight decline in free radical scavenging ability in kiwiberry fruits stored for 7 days at 1 °C.

In this study, the antiradical activity measured by the ABTS and CUPRAC methods was significantly lower in the fruits stored under controlled atmosphere (CA) conditions (Table 5). Similar findings have been reported for blueberries and raspberries, where long-term CA storage led to modifications in phenolics and reduced activity in the ABTS and DPPH assays [60]. It is plausible that the low oxygen levels in CA alter the activity of oxidative enzymes, resulting in unfavorable transformations of phenolic compounds. Conversely, studies on yellow peaches by Dong et al. [61] showed that the fruits stored in CA exhibited a significantly higher antiradical activity, measured by the DPPH, FRAP, and ABTS methods, compared with storage in normal air. While CA is widely recognized as the most effective method for inhibiting ethylene synthesis, it also influences ascorbic acid losses [22], a phenomenon confirmed by the results of the present study.

A decrease in reducing capacity during cold storage, measured by the FRAP method—which relies on the SET mechanism at an acidic pH—was also reported by Tavarini et al. [62] in ‘Hayward’ kiwifruit, despite an increased phenolic content. Similar findings were reported by Krupa et al. in hardy kiwifruits [51]. Tavarini et al. [62] suggested that vitamin C contributes more substantially to the antioxidant capacity of kiwifruit than other antioxidants like phenolics or carotenoids. The current study, which employed a broader range of antioxidant-mechanism assays beyond the commonly used ABTS, DPPH, and reducing capacity tests, refines this suggestion. We observed a statistically significant decline in ascorbate content during storage, which generally correlated with a marked decrease in antiradical activity measured by methods based primarily on the SET mechanism (CUPRAC, ABTS). The course of changes in the activity and content of ascorbate allows us to suggest that the SET-based antiradical activity in kiwifruit depends on the content of ascorbate and probably also on the status of thiol antioxidants, mainly glutathione. In this work, the total content of both groups of compounds was determined, which includes the oxidized and reduced forms. This is probably the reason for the lack of unequivocal coverage of activity changes in the determinations of these components. Phenolics exhibited clearly different behaviors. Their content may explain the relatively stable antiradical activity observed with the HAT-based CBA method.

Moreover, the data suggest an intriguing compensatory effect, where the loss of antioxidant activity based on the SET mechanism is balanced by an increased capacity to chelate transition metal ions, potentially supported by favorable trends in available phenolics’ chemical structure. Transition metal binding plays a crucial role in inhibiting free radical lipid oxidation, during which the behavior of antioxidants is much better reflected by basic research, according to the HAT mechanism. This is why testing antioxidants using not only methods relying on the SET mechanism, but also according to the HAT mechanism, is important for a good overview of their potential.

### 2.4. Multivariate PCA Biplot Assessment

**Figure 5:**
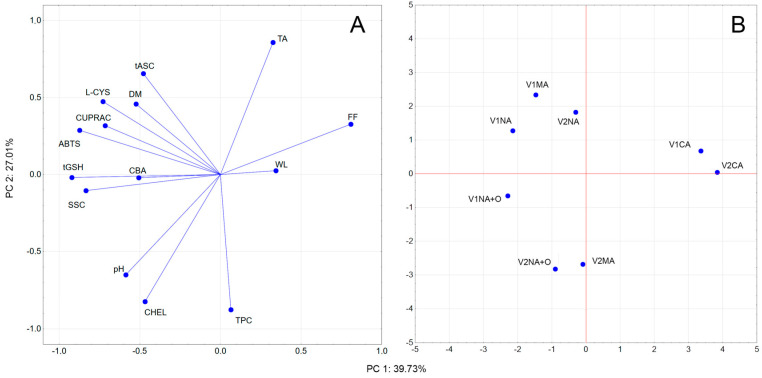
PCA biplot (PC1 vs. PC2) for four storage combinations and two pre-storage treatments examined and their physicochemical and antioxidant properties. (**A**) Projection of variables (Traits vs. Treatments_ onto the plane of cases, (**B**) Treatments and Variants. Abbreviations, CUPRAC assay; ABTS assay; Fe(II) chelating activity (CHEL); crocin bleaching assay (CBA); total ascorbate (tASC); L-cysteine (L-CYS); glutathione (tGSH); total phenolic content (TPC); soluble solid content (SSC); titratable acidity (TA); pH; dry matter (DM); weight loss (WL); firmness (FF); Designations: V1—postharvest variant with storage at 8 °C for 14 h (pre-cooling), V2—postharvest variant without pre-cooling after harvest, NA—normal air, MA—modified atmosphere, NA+O normal air + ozone, CA—controlled atmosphere.

To assess the interdependence of the tested storage combinations, pre-storage treatments, and factors, a multivariate principal component analysis (PCA) based on the average data for all terms of storage was performed. The projection of variable factors responsible for 66.74% of the variability indicates the positive interdependence of DM, tASC, L-CYS, and ABTS (first group) and CUPRAC, pH, TPC, and CHEL (second group), as well as FF, TA, and WL (third group) (Figure 5A).

The first group of factors is located in different but adjacent quadrants of the chart, suggesting a lack of a significant relationship between this and the other two groups. However, groups two and three are located in opposite quadrants, suggesting their negative mutual dependence.

A plot of the projection of cases onto the plane of factors allows us to group the studied storage combinations, pre-storage treatments based on their mutual similarity and differences, and their relationship to the studied factors. As shown in Figure 5B, combinations V1MA, V2NA, and V1NA were positively correlated with DM, tASC, L-CYS, CUPRAC, and ABTS. In turn, V2NA+O, V2MA were positively correlated with the TPC, pH, and CHEL and negatively with titratable acidity (TA) and firmness (FF). And finally, V1CA and V2CA were positively correlated with FF. V1NA+O showed no strong affinity for the tested factors.

Pre-cooling the samples did not affect the results obtained for individual variants, with the exception of storage in a modified atmosphere. The PC2 variable differentiated them, primarily influenced by the TPC and the ability to chelate metal ions. Considering all variants, we found that the samples stored in a controlled atmosphere differed from the other storage treatments. The PC1 variable differentiated them primarily by FF and WL (positively correlated with the CA storage and negatively with the other treatments), as well as CBA, tGSH, SSC, and to a lesser extent ABTS and CUPRAC (negatively correlated with the CA and positively with the other treatments).

## 3. Materials and Methods

### 3.1. Fruit Samples, Weather Conditions, and Experimental Design

The experiment was conducted in 2021 using fruit from the Weiki variety, characterized by an average fruit weight of 8–13 g, green skin with a prominent cherry-red blush, and a cylindrical, slightly laterally flattened shape. The fruits were randomly harvested from a commercial two-hectare plantation located in Mazovian Voivodeship, Poland (51.7972166 N, 20.8120578 E) at the harvest maturity stage (firm fruit, SSC 6.5–7%).

The 2021 season was favorable for *A. arguta* growth and fruiting. The average annual temperature was 9.3 °C, and the total rainfall was 885.2 mm. Both the temperature and total rainfall exceeded the multi-year average for 1981–2010 (8.9 °C and 574.5 mm, respectively). The highest rainfall was recorded during the growing season, especially in May, July, and August (Table 6). This specific distribution of precipitation, along with the higher average temperatures in summer, was beneficial for this cultivation.

**Scheme 1:**
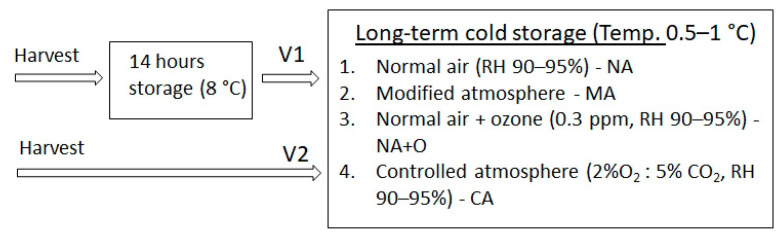
Experiment layout.

The study included two post-harvest treatment variants: V1, where the fruit was stored overnight at about 8 °C (14 h) and packed the next day at roughly 17 °C (a common practice on kiwiberry farms), and V2, where the fruit was packed and placed directly into cold storage on the day of harvest. Cold-storage conditions consisted of four treatments: 1. normal air, temp. of 0.5–1 °C with a relative humidity (RH) of 90–95% (NA); 2. modified atmosphere storage using Xtend^®^ bags in a common cold store, temp. of 0.5–1 °C (MA); 3. normal air with continuous ozonation at 0.3 ppm (NA+O), temp. of 0.5–1 °C with a relative humidity (RH) of 90–95%; and 4. controlled atmosphere storage with 2% O_2_ and 5% CO_2_, temp. of 0.5–1 °C with a RH of 90–95% (CA). Each storage setup was equipped with an ethylene scavenger. The storage experiments for both variants started on the same day. All procedures took place at the grower’s cooling facilities, which included an Atom II Mix 80 ozone generator managed by an OS 6 controller (Blue Planet, Wolsztyn, Poland), an Aero 5s air humidifier (Otech, Żory, Poland), and ethylene-absorbing filters. The experimental setup is illustrated in Scheme 1.

The fruits were packed in standard PET punnets, weighing approximately 500 g each, then weighed and grouped into sets of four inside plastic boxes measuring 30 × 40 cm. For the modified atmosphere (MA) treatment, the four PET punnets were first sealed together in Xtend^®^ MA packaging dedicated to kiwiberries (StePac L.A. Ltd., Tefen, Israel) before being placed into the boxes. In this setup, the entire box served as the experimental replicate. The first analyses were conducted on the fruit before being placed in cold storage (Term 0). Subsequent fruits from each storage combination were removed from the cold storage every 10 days (Terms 1–5). For each sampling date and storage condition, around 2 kg of fruit (one box) was collected. However, for the MA treatment, three boxes (each in a separate MA package) were sampled per date; each box with four punnets represented one replicate. Before storage and at each sampling point after removal from cold storage, the fruits were weighed and subjected to basic physicochemical analyses, including for firmness (FF), soluble solids content (SSC), titratable acidity (TA), pH, and dry matter content (DM). About 600 g of fruit (three samples of 200 g each) were frozen in liquid nitrogen and then freeze-dried. The freeze-dried samples were stored under cold conditions until further chemical analysis. On each sampling date, the fruits were visually inspected immediately after removal from storage to assess their health. Any presence of spoiled fruits led to the disqualification of that batch from further storage and analysis.

### 3.2. Basic Fruit Physicochemical Parameters

Fruit weight loss was calculated as the difference between the fruit weight at the time of placement into cold storage and its weight immediately after removal at each sampling date. Firmness was measured using a Texture Analyzer TX-700 (Lamy Rheology, Champagne au Mont d’Or, France). Measurements were taken perpendicular to the narrower width of the fruit, including the skin, using a 6 mm probe at a speed range of 0.1 to 10 mm/s ± 0.2%. Results were expressed in Newtons (N). Dry matter content (DM) was determined gravimetrically following the AOAC method (2000) [63]. About 1.0 g of freeze-dried sample was accurately weighed to 0.0001 g precision using a laboratory balance. The samples were then dried in a laboratory dryer (SUP 200W, Wamed, Warsaw, Poland) at 105 °C until reaching a constant weight. The DM was calculated from the mass difference before and after drying and expressed as a percentage (%).

Physicochemical tests, such as the total soluble solids, pH, and total (titratable) acidity, were performed on samples of fresh material collected from individual storage combinations at 10-day intervals throughout the storage period. The tests were conducted on fresh juice obtained with a slow juicer (Zelmer Lumiere ZJE 1900X, Zelmer S.A., Warsaw, Poland). The total extracted content was determined using a refractometric method with an Abbe refractometer (ORT-1, Kern & Sohn GmbH, Balingren-Frommern, Germany) according to PN-EN 12143:2000 [64], with results expressed in percent. The titratable (total) acidity was measured by a titrimetric method following PN-EN 12147:2000 [65], which involves neutralizing the acids in the tested solution through titration with sodium hydroxide, using phenolphthalein as an indicator. The results were expressed in grams per 100 grams of fresh weight (g/100 g FW). The pH measurements were performed using a potentiometric method on freshly prepared juices with a pH meter (Elmetron CP-511, Elmetron G.P., Zabrze, Poland). For this, approximately 20 mL of the sample was transferred to a 50 mL glass beaker, the probe was immersed, and, after allowing the reading to stabilize at room temperature (20 °C), the pH value was recorded.

### 3.3. Fruit Chemical Analyses

The fruits for chemical analyses were taken from the cold storage and portioned at 200–300 g per sample, then subjected to freeze-drying. Freeze-drying was performed in a laboratory freeze-dryer Alfa 1-4 LSC (Donserv, Warsaw, Poland), with a chamber temperature of −50 °C, pressure of 10 Pa, and a shelf temperature of 21 °C for 120 h. After drying, the material was weighed, ground in a laboratory mill (Grandomix GM 200, Retsch, Katowice, Poland), then placed in sterile plastic containers with screw caps, tightly sealed, and stored in a freezer (Arctiko, A/S, Esbjerg N, Denmark) at −20 °C until chemical analyses and antioxidant activity testing.

Total ascorbate (tASC = AA + DHA—the sum of reduced (ascorbic acid, AA) and oxidized forms (dehydroascorbic acid, DHA)) and thiol compounds (L-cysteine, L-CYS, and total glutathione—the sum of reduced (GSH) and oxidized (GSSG)) were measured using a HPLC technique (Waters Co., Milford, MA, USA). A total of 0.1 molar hydrochloric acid was used to extract the compounds. Thiol compounds, after being converted to fluorescent derivatives with monobromobimane, were determined using a fluorescence detector at 480 nm by excitation at 380 nm on an X-Bridge C18 column (250 mm, 4.6 mm, 5 µm, Waters Co., Milford, MA, USA) by applying a solution of 10% methanol containing 0.25% (*v*/*v*) glacial acetic acid (solvent A, pH 4.3) and 90% methanol with the same concentration of acetic acid (solvent B, pH 3.9); the flow rate was 1 mL min^−1^. Ascorbate, after being oxidized from L-AA to dehydroascorbate with ascorbate oxidase and derivatization with o-phenylenediamine, was detected as a fluorescent compound at 350 nm by excitation at 450 nm under isocratic conditions. The eluent used was 20% methanol with 800 mM K_2_HPO_4_ (pH 7.8) at a flow rate of 1 mL min^−1^. The column was the same column as that for thiol compounds. Results were calculated using a standard curve and commercially available compounds (L-ascorbic acid, glutathione, and L-cysteine) [54].

The TPC (total phenolic content) was measured using a modified colorimetric spectrophotometric method following Singleton and Rossi [66] with the Folin–Ciocalteu reagent. For sample extraction, 0.5 g (with an accuracy of 0.001 g) of lyophilized research material was weighed on an analytical balance (AS 220/X, Radwag, Radom, Poland) into sterile plastic Falcone-type test tubes with screw caps (50 mL capacity), and 30 mL of 80% aqueous methanol solution (PPH Stanlab Sp. z o.o., Lublin, Poland) was added. The samples were shaken in a Vortex shaker (Wizard Advanced IR Vortex Mixer, VELP Scientifica Srl, Usmate (MB), Italy) for 60 s at 2000 rpm to ensure thorough mixing. Then, they were incubated in a shaking incubator (IKA KS 4000i Control, IKA^®^ Polska Sp. z o. o., Warsaw, Poland) for 60 min at 60 °C and 200 rpm. After incubation, the samples were shaken again in a Vortex shaker for 60 s, then centrifuged in a refrigerated centrifuge (MPW Med. Instruments, Warsaw) for 15 min at 4 °C and 10,000 rpm. The supernatant obtained was used to determine the TPC. To express the TPC, a calibration curve was prepared using gallic acid as the standard, and the absorbance measurements of the extracts were expressed as g GAE per kilogram of dry weight (DW).

### 3.4. Determination of In Vitro Antioxidant/Antiradical Activities

#### 3.4.1. Preparation of Methanolic Extract and Method of Measurements

A total of 500 mg of lyophilizate was weighed into centrifuge tubes, to which 30 mL of an 80% methanol solution was then added. The suspension was stirred for 30 s using a vortex mixer, after which the tubes were placed in an ultrasonic bath at 40 °C for 20 min. The tubes were then centrifuged for 20 min at 4 °C and 10,000 rpm. The supernatant was stored at −18 °C for further analysis. All extracts were prepared in triplicate. Antioxidant activity was measured using three different methods: the ABTS assay [67], CUPRAC assay [68], and CBA [69,70]. Fe(II) chelating activity was determined by the method described by Lai et al. [71].

#### 3.4.2. ABTS^•+^ Assay

ABTS^•+^ was prepared following the original method of Re et al. [67] by mixing equal volumes of substrate solution (2,2′-azino-bis(3-ethylbenzothiazoline-6-sulfonic acid, 7 mM) and oxidant (potassium persulfate, 2.45 mM). The radicals were generated in the dark for 24 h, then the solution was diluted with 96% ethanol until reaching an absorbance of 0.7 ± 0.02 at 734 nm. Next, 40 μL of the extract was added to the tubes, followed by 4 mL of the diluted ABTS solution. The mixture was stirred, and after 6 min, the absorbance was measured against a blank containing 40 μL of extract and 4 mL of ethanol. A calibration curve for Trolox concentrations ranging from 0 to 16 μg/mL was prepared. The results were expressed as mg of Trolox per gram of DW. The analysis was performed in triplicate.

#### 3.4.3. CUPRAC Assay

To the tubes, 1 mL of 10 mM copper(II) chloride solution, 1 mL of 7.5 mM neocuproine solution, and 1 mL of 1 M ammonium acetate buffer (pH 7.0) were added, followed by 0.1 mL of extract and 1 mL of 80% methanol. After mixing the entire mixture, the absorbance was measured at 450 nm against a reagent blank after 30 min. A calibration curve was plotted for Trolox concentrations ranging from 8 to 80 μg/mL. The results were expressed as mg Trolox per gram of DW. Analyses were performed in triplicate.

#### 3.4.4. CBA

A 1 mg/mL methanolic stock solution of crocin (Sigma-Aldrich, Merck Life Science, Poznań, Poland) was prepared and stored at 4 °C. For analysis, the solution was diluted with PBS buffer to make a 12 mg/mL solution. Two milliliters of this solution were transferred to tubes, followed by the addition of 0.2 mL of extract and 0.8 mL of 80% methanol. The tubes were shaken in a 37 °C water bath for 5 min. The oxidation reaction was initiated by adding 1 mL of a 0.8% AAPH (2,2′-azobis(2-methylpropionamidine) dihydrochloride) solution in PBS, preheated to 37 °C. After 75 min, the decrease in crocin absorbance at 443 nm was measured. Blank and control samples containing crocin, methanol, and either 80% methanol or AAPH with PBS were also prepared. The activity of the samples was expressed relative to the control [%] and then converted to mg Trolox/g DW based on the Trolox activity measured under the same conditions.

#### 3.4.5. Fe(II) Chelating Activity

In total, 0.75 mL of each extract was placed in a test tube. Then, 1.75 mL of 80% methanol and 50 μL of a 2 mM FeCl_2_ solution were added. The mixture was left to stand for 20 min. Then, 0.5 mL of a five mM ferrozine solution (3-(2-pyridyl)-5,6-diphenyl-1,2,4-triazine-4,4’-disulfonic acid sodium salt) was added. After 10 min, the absorbance of the resulting colored complex was measured at 562 nm. A calibration curve was plotted for iron(II) concentrations in the range 0–0.15 μM. The results were expressed as μM Fe(II)/g DW Analyses were performed in triplicate.

### 3.5. Statistical Analysis

All assays were performed in triplicate, and the results were presented as mean ± standard deviation (SD) (*n* = 3). Three-way ANOVA test, followed by the HSD Tukey post–hoc procedure at *p* = 0.05, and the multivariate principal component analysis (PCA) biplot method were employed to analyze data through STATISTICA version 13.0 software (TIBCO Software Inc., Santa Clara, CA, USA). A summary of the statistical analysis is presented in Table 3.

## 4. Conclusions

The *Actinidia arguta* fruit (kiwiberry) has a limited storage capacity. Therefore, there is a continuing need to explore various solutions and options that would be useful for storage. In our study, eight variants of cold storage were tested, differing in terms of conditions (NA, MA, NA+O, CA with or without pre-cooling) with respect to the fruit’s physicochemical quality. Irrespective of refrigeration conditions, the fruit’s firmness and acidity decreased rapidly, while the soluble solid content increased. These changes occur most slowly under controlled or modified atmosphere conditions (Xtend^®^ bags). The conditions and duration of fruit storage significantly affected the content and metabolism of the tested antioxidants. Low-molecular-weight hydrophilic antioxidants such as ascorbate, glutathione, and its precursor L-cysteine, underwent considerable fluctuations during storage. Our results indicate that a high antioxidant content can be expected within the first ten days of fruit storage, which is the result of the fruit’s adaptation strategy to new environmental conditions, in which antioxidants play a significant role. Phenolic compounds were the most stable in terms of content in the *A. arguta* fruit. However, the observed picture of changes in the content of biologically active compounds can be used depending on the intended use of the fruit, i.e., consumption purposes, processing, or other industry.

Changes in the antiradical activity of stored fruit were highly complex and depended on the reaction mechanism used to assess it. Using only tests based on the single electron transfer (SET) mechanism does not provide a complete picture, and it is also necessary to employ methods based on the hydrogen atom transfer (HAT) mechanism. Changes in fruit components that are unfavorable from the SET perspective may, paradoxically, result in improvements in other antioxidant-related properties.

There was no clear effect of fruit pre-cooling on their overall perceived antioxidant activity, as this treatment affected the level of individual antioxidants in different ways. When considering storage methods applied in our study, using any of the three alternatives to cold storage in CA (NA, NA+O, MA) proved beneficial in short-term storage. However, MA has proven to be a similarly effective long-term storage method to CA in terms of the physicochemical quality of the *A. arguta* fruit. The positive effects of ozonation at different concentrations and varieties require further verification.

## Figures and Tables

**Table 1:** The average water lost (WL) and firmness (FF) values for the tested main factors: cooling effect in the column (V1 vs. V2) and storage method in the row (NA, MA, NA+O, CA).

Variant	NA	MA	NA+O	CA	Average
**WL**					
V1	2.1b	0.2a	2.1b	2.4cd	1.7A
V2	2.1b	0.2a	2.2bc	2.5d	1.8A
Average	2.1B	0.2A	2.2B	2.5C	
**FF**					
V1	31.6bc	31.9c	25.3a	34.8d	30.9A
V2	31.2bc	30.8bc	29.6b	35.9d	31.9B
Average	31.4B	31.4B	27.5A	35.3C	

Individual data for WL and FF marked with the same capital letter for mean values, separately in the column and in the row, and separately marked with the same lower-case letter for variants and combinations are not significantly different (Tukey HSD test at *p* ≤ 0.05).

**Table 2:** The average soluble solids (SSC), titratable acidity (TA), pH and dry matter content (DM) values for the tested main factors: cooling effect in the column (V1 vs. V2) and storage method in the row (NA, MA, NA+O, CA).

Variant	NA	MA	NA+O	CA	Average
**SSC**					
V1	10.64d	10.22bc	10.69d	10.00b	10.39B
V2	10.00b	10.36cd	10.14bc	9.64a	10.03A
Average	10.32B	10.29B	10.42B	9.82A	
**TA**					
V1	1.08bc	1.07bc	1.03ab	1.11c	1.07B
V2	1.07bc	0.98a	0.99a	1.06bc	1.02A
Average	1.08B	1.02A	1.01A	1.08B	
**pH**					
V1	3.62abc	3.58ab	3.67d	3.60ab	3.62A
V2	3.58ab	3.63bcd	3.66cd	3.57a	3.61A
Average	3.60A	3.61A	3.66B	3.58A	
**DM**					
V1	17.4c	17.0abc	17.3bc	17.0abc	17.2B
V2	17.3bc	17.1abc	16.7a	16.8ab	16.9A
Average	17.3B	17.1AB	17.0A	16.9A	

Individual data for SSC, TA, pH and DM marked with the same capital letter for mean values, separately in the column and in the row, and separately marked with the same lower-case letter for variants and combinations are not significantly different (Tukey HSD test at *p* ≤ 0.05).

**Table 3:** Summary of analysis of variance (ANOVA) for components tested; values of F for particular sources of variation and their significance.

Component	Source of Variation													
	Pre-Storage Variant ^1^ (A)		Cold Storage Conditions ^2^ (B)		Time of Storage ^3^ (C)		Interactions							
							AB		AC		BC		ABC	
df	1		3		5		3		5		15		15	
WL	3.46	ns	913.1	***	965.3	***	1.23	ns	4.75	***	91.26	***	1.48	ns
FF	7.75	***	89.17	***	1162.3	***	12.59	***	13.01	***	28.38	***	6.02	***
DM	6.9	*	5.5	**	40.5	***	3.1	*	4.6	***	3.5	***	5.8	***
SSC	39.4	***	22.4	***	1104.4	***	9.6	***	3.5	**	5.1	***	11.2	***
TA	17.86	***	11.79	**	739.7	***	1.9	ns	4.54	***	4.51	ns	2.81	**
pH	1.3	ns	18.5	***	274.2	***	5.1	**	4.8	***	6.1	***	7.9	ns
TPC	506.2	***	52.6	***	460.1	***	57.3	***	77.6	***	12.6	***	19.0	***
tASC	3.34	ns	0.78	ns	30.86	***	3.78	*	1.94	ns	4.79	***	1.59	ns
tGSH	2.22	ns	16.75	***	32.55	***	2.22	ns	7.81	***	2.79	**	3.63	***
L-CYS	11.04	**	3.77	*	63.64	***	1.30	ns	2.38	*	2.21	*	3.81	***
ABTS	5.15	*	7.72	***	356.0	***	2.87	*	13.82	***	2.36	*	0.59	ns
CUPRAC	0.32	ns	6.62	***	266.72	***	1.33	ns	25.91	***	1.59	ns	0.94	ns
CBA	17.87	***	10.40	***	15.93	***	6.51	***	7.00	***	3.56	***	1.88	*
CHEL	0.04	ns	7.93	***	166.34	***	4.57	**	5.58	***	2.47	**	3.48	***

*** significant at *p* = 0.001; ** significant at *p* = 0.01; * significant at *p* = 0.05; ns—non-significant. ^1^ Variant with pre-storage cooling up to 8 °C and without cooling; ^2^ normal air, normal air + ozone, modified atmosphere, controlled atmosphere; ^3^ five appointments every 10 days started from 16 September 2021. Abbreviations: df—degrees of freedom; WL—weight lost; FF—firmness; DM—dry matter content; SSC—soluble solid content; TA—titratable acidity; TPC—total phenolic content; tASC—total ascorbate content; tGSH—total glutathione content; L-CYS—L-cysteine content; ABTS antiradical assay; CUPRAC antiradical assay; CBA—crocin bleaching assay; CHEL—Fe(II) chelating activity.

**Table 4:** The average ascorbate (tASC), L-cysteine (L-CYS), glutathione (tGSH) and total phenolics content (TPC) values for the tested main factors: cooling effect in the column (V1 vs. V2) and storage method in the row (NA, MA, NA+O, CA).

Variant	NA	MA	NA+O	CA	Average
**tASC**					
V1	1.70a	1.93a	1.89a	1.59a	1.78A
V2	1.83a	1.49a	1.61a	1.66a	1.65A
Average	1.76A	1.72A	1.75A	1.62A	
**L-CYS**					
V1	6.96b	7.78b	6.12ab	5.69ab	6.64B
V2	5.93ab	5.44ab	5.87ab	4.22a	5.37A
Average	6.45B	6.61B	5.99AB	4.96A	
**tGSH**					
V1	271c	246c	237bc	184ab	236A
V2	238bc	222bc	265c	162a	222A
Average	257A	234B	252B	173A	
**TPC**					
V1	7.41ab	7.04a	7.43b	7.21ab	7.27Aa
V2	7.48b	8.21d	8.63e	7.92c	8.06B
Average	7.45A	7.63B	8.03C	7.56AB	

Individual data for tASC, L-CYS, tGSH and TPC marked with the same capital letter for mean values, separately in the column and in the row, and separately marked with the same lower-case letter for variants and combinations are not significantly different (Tukey HSD test at *p* ≤ 0.05).

**Table 5:** The average CUPRAC, ABTS, Fe(II) chelating activity (CHEL) and CBA values for the tested main factors: cooling effect in the column (V1 vs. V2) and storage method in the row (NA, MA, NA+O, CA).

Variant	NA	MA	NA+O	CA	Average
**CUPRAC**					
V1	20.08b	19.54ab	18.61ab	16.39a	18.65A
V2	20.64b	18.16ab	19.16ab	17.83ab	18.95A
Average	20.36B	18.85AB	18.88AB	17.11A	
**ABTS**					
V1	8.84b	8.81b	8.04ab	6.86a	8.14B
V2	7.89ab	7.32a	8.24ab	6.92a	7.59A
Average	8.36B	8.07B	8.14B	6.89A	
**CHEL**					
V1	0.68abc	0.58abc	0.83bc	0.56abc	0.66A
V2	0.42a	0.93c	0.83c	0.46ab	0.67A
Average	0.55AB	0.76BC	0.85C	0.51A	
**CBA**					
V1	14.46b	14.49b	14.15b	12.10a	13.80A
V2	15.27b	14.21b	14.84b	14.44b	14.69B
Average	14.86B	14.35B	14.50B	13.27A	

Individual data for CUPRAC, ABTS, CHEL and CBA marked with the same capital letter for mean values, separately in the column and in the row, and separately marked with the same lower-case letter for variants and combinations are not significantly different (Tukey HSD test at *p* ≤ 0.05).

**Table 6:** Meteorological data for the 2021 season.

2021	January	February	March	April	May	June	July	August	September	October	November	December
Average temperature (°C)	−1.1	−1.8	3.8	7.4	13.2	20.8	22.4	17.6	14.1	9.8	5.6	−0.7
Sum of precipitation (mm)	42.9	42.4	17.4	65	110.2	73.3	201.1	203.1	42	10.2	48.9	28.7

## Data Availability

Data are contained within the article.

## References

[1] Musacchi S., Serra S. (2018). Apple Fruit Quality: Overview on Pre-Harvest Factors. Sci. Hortic..

[2] Tiwari U., Cummins E. (2013). Factors Influencing Levels of Phytochemicals in Selected Fruit and Vegetables during Pre- and Post-Harvest Food Processing Operations. Food Res. Int..

[3] Mditshwa A., Magwaza L.S., Tesfay S.Z., Mbili N. (2017). Postharvest Quality and Composition of Organically and Conventionally Produced Fruits: A Review. Sci. Hortic..

[4] Liang X., Qian G., Pan S., Wang J., Cong X., Ye T., Yan M., Xu H., Xin G. (2024). A Shelf Life Prediction Model of *Actinidia arguta* ‘Chang Jiang No.1’ Based on Postharvest Quality Evaluation Combined with Fuzzy Mathematics. J. Stored Prod. Res..

[5] Wang Y., Xu F., Feng X., MacArthur R.L. (2015). Modulation of *Actinidia arguta* Fruit Ripening by Three Ethylene Biosynthesis Inhibitors. Food Chem..

[6] Fisk C.L., McDaniel M.R., Strik B.C., Zhao Y. (2006). Physicochemical, Sensory, and Nutritive Qualities of Hardy Kiwifruit (*Actinidia arguta* ‘Ananasnaya’) as Affected by Harvest Maturity and Storage. J. Food Sci..

[7] Figiel-Kroczyńska M., Ochmian I. (2021). Effect on Phytochemical Content and Microbial Contamination of Actinidia Fruit after Shock Cooling and Storage. Acta Univ. Cibiniensis. Ser. E Food Technol..

[8] Stefaniak J., Stasiak A., Latocha P., Łata B. (2019). Seasonal Changes in Macronutrients in the Leaves and Fruit of Kiwiberry: Nitrogen Level and Cultivar Effects. Commun. Soil Sci. Plant Anal..

[9] Latocha P., Łata B., Jankowski P. (2023). Variation of Chemical Composition and Antioxidant Properties of Kiwiberry (*Actinidia arguta*) in a Three-Year Study. Molecules.

[10] Wannemuehler S.D., Luby J.J., Yue C. (2023). Consumer Preferences for Kiwiberries: Implications of Experimental Auctions. HortScience.

[11] Macedo C., Silva A.M., Ferreira A.S., Cádiz-Gurrea M.d.l.L., Fernández-Ochoa Á., Segura-Carretero A., Delerue-Matos C., Costa P., Rodrigues F. (2023). Insights into the Polyphenols Extraction from *Actinidia arguta* Fruit (Kiwiberry): A Source of pro-Healthy Compounds. Sci. Hortic..

[12] Garcia-Herrera P., Maieves H.A., Vega E.N., Perez-Rodriguez M.L., Fernandez-Ruiz V., Iriondo-DeHond A., Castillo M.D.d., Sanchez-Mata M.C. (2022). Dwarf Kiwi (*Actinidia arguta* Miq.), a Source of Antioxidants for a Healthy and Sustainable Diet. Molecules.

[13] Zhang H., Teng K., Zang H. (2023). *Actinidia arguta* (Sieb. et Zucc.) Planch. Ex Miq. A Review of Phytochemistry and Pharmacology. Molecules.

[14] Latocha P., Debersaques F., Decorte J. (2015). Varietal Differences in the Mineral Composition of Kiwiberry—*Actinidia arguta* (Siebold Et Zucc.) Planch. Ex. Miq. Acta Hortic..

[15] Cotrut R., Udriste A. (2017). A Review of How to Optimize Storage and Shelf Life Extending Technology of Kiwifruit (*Actinidia* Sp.) by Using 1-Methylcyclopropene to Measurably Reduce Fruit Waste. Sci. Pap.—Ser. B Hortic..

[16] White A., Nihal de Silva H., Requejo-Tapia C., Roger Harker F. (2005). Evaluation of Softening Characteristics of Fruit from 14 Species of *Actinidia*. Postharvest Biol. Technol..

[17] Xiong S., Zhou F., Jiang A., Yang L., Hu W. (2024). Ethanol Vapor Ameliorates Chilling Injury and Maintains Postharvest Quality by Increasing Antioxidant Capacity of Hardy Kiwifruit (*Actinidia arguta*). Sci. Hortic..

[18] Wang T., Sui Y., Du X., Zhang S., Chen L. (2025). A Comprehensive Review of Post-Harvest Ripening, Preservation and Processing for *Actinidia arguta* (Mini Kiwi). J. Stored Prod. Res..

[19] Eo H.J., Kim C.-W., Lee U., Kim Y. (2024). Comparative Analysis of the Characteristics of Two Hardy Kiwifruit Cultivars (*Actinidia arguta* Cv. Cheongsan and Daebo) Stored at Low Temperatures. Plants.

[20] Xia Y., Wu D.-T., Ali M., Liu Y., Zhuang Q.-G., Wadood S.A., Liao Q.-H., Liu H.-Y., Gan R.-Y. (2024). Innovative Postharvest Strategies for Maintaining the Quality of Kiwifruit during Storage: An Updated Review. Food Front..

[21] Jeong H.-R., Cho H.-S., Cho Y.-S., Kim D.-O. (2020). Changes in Phenolics, Soluble Solids, Vitamin C, and Antioxidant Capacity of Various Cultivars of Hardy Kiwifruits during Cold Storage. Food Sci. Biotechnol..

[22] Paulauskienė A., Tarasevičienė Ž., Žebrauskienė A., Pranckietienė I. (2020). Effect of Controlled Atmosphere Storage Conditions on the Chemical Composition of Super Hardy Kiwifruit. Agronomy.

[23] Krupa T., Tomala K. (2021). Effect of Oxygen and Carbon Dioxide Concentration on the Quality of Minikiwi Fruits after Storage. Agronomy.

[24] Krupa T., Klimek K., Zaraś-Januszkiewicz E. (2022). Nutritional Values of Minikiwi Fruit (*Actinidia arguta*) after Storage: Comparison between DCA New Technology and ULO and CA. Molecules.

[25] Fisk C.L., Silver A.M., Strik B.C., Zhao Y. (2008). Postharvest Quality of Hardy Kiwifruit (*Actinidia Arguta* ‘Ananasnaya’) Associated with Packaging and Storage Conditions. Postharvest Biol. Technol..

[26] Stefaniak J., Sawicka M., Krupa T., Latocha P., Łata B. (2017). Effect of Kiwiberry Pre-Storage Treatments on the Fruit Quality during Cold Storage. Zemdirbyste-Agriculture.

[27] Xu D., Zhou F., Gu S., Feng K., Hu W., Zhang J., Sun X., Liang X., Jiang A. (2021). 1-Methylcyclopropene Maintains the Postharvest Quality of Hardy Kiwifruit (*Actinidia arguta*). Food Meas..

[28] Xiong S., Sun X., Tian M., Xu D., Jiang A. (2023). 1-Methylcyclopropene Treatment Delays the Softening of *Actinidia arguta* Fruit by Reducing Cell Wall Degradation and Modulating Carbohydrate Metabolism. Food Chem..

[29] Wang Y., Niu Y., Ye L., Shi Y., Luo A. (2023). Ozone Treatment Modulates Reactive Oxygen Species Levels in Kiwifruit through the Antioxidant System: Insights from Transcriptomic Analysis. J. Plant Physiol..

[30] Piechowiak T., Grzelak-Błaszczyk K., Sójka M., Balawejder M. (2022). One-Time Ozone Treatment Improves the Postharvest Quality and Antioxidant Activity of *Actinidia arguta* Fruit. Phytochemistry.

[31] Guerreiro A.C., Gago C., Passos D., Martins J., Cruz S., Veloso F., Guerra R., Antunes M.D. (2025). Edible Coatings Enhance Storability and Preserve Quality of Kiwiberry (*Actinidia arguta* L.) Cv. Ken’s Red. Horticulturae.

[32] Wang J., Qian G., Pan S., Ye T., Yan M., Liang X., Hui L., Cong X., Yang R., Xu H. (2023). Evaluation Storage Capacity of Six Kind Late-Maturing *Actinidia arguta* Resources. J. Stored Prod. Res..

[33] Zheng Q., Tian W., Wang S., Chen Z., Wang H., Yue L., Yan W., Qi W., Zhang C., Xu X. (2025). Electron Beam Irradiation Maintains Postharvest Quality of *Actinidia arguta* by Regulating the Cell Wall, Starch Degradation, and Antioxidant Capacity. Postharvest Biol. Technol..

[34] Hui L., Pan S., Qian G., Yan M., Li Y., Yang R., Ye T., Liang X., Cong X., Xu H. (2025). Postharvest Short-Time Partial Dehydration Extends Shelf-Life and Improves the Quality of *Actinidia arguta* during Low Temperature Storage. J. Future Foods.

[35] Ying L., Bian J., Zhao F., Chen X., Tang J., Jiang F., Sun B. (2024). Short-Term Anaerobic Treatment Maintained the Quality of *Actinidia arguta* by Activating the Antioxidant Defense System. J. Sci. Food Agric..

[36] Pathare P.B., Rahman M.S. (2022). Nondestructive Quality Assessment Techniques for Fresh Fruits and Vegetables.

[37] Yu Y., Yao M. (2022). A Portable NIR System for Nondestructive Assessment of SSC and Firmness of Nanguo Pears. LWT.

[38] Latocha P., Krupa T., Jankowski P., Radzanowska J. (2014). Changes in Postharvest Physicochemical and Sensory Characteristics of Hardy Kiwifruit (*Actinidia arguta* and its Hybrid) after Cold Storage under Normal versus Controlled Atmosphere. Postharvest Biol. Technol..

[39] Han Y., East A., Nicholson S., Jeffery P., Glowacz M., Heyes J. (2022). Benefits of Modified Atmosphere Packaging in Maintaining ‘Hayward’ Kiwifruit Quality at Room Temperature Retail Conditions. N. Z. J. Crop Hortic. Sci..

[40] Brizzolara S., Manganaris G.A., Fotopoulos V., Watkins C.B., Tonutti P. (2020). Primary Metabolism in Fresh Fruits During Storage. Front. Plant Sci..

[41] Sarron E., Gadonna-Widehem P., Aussenac T. (2021). Ozone Treatments for Preserving Fresh Vegetables Quality: A Critical Review. Foods.

[42] Li X., Zeng Y., Wang T., Jiang B., Liao M., Lv Y., Li J., Zhong Y. (2024). Dynamic Analysis of the Fruit Sugar-Acid Profile in a Fresh-Sweet Mutant and Wild Type in ‘Shatangju’ (*Citrus Reticulata* Cv.). Plants.

[43] Giuggioli N.R., Briano R., Baudino C., Peano C. (2019). Post-Harvest Warehouse Management of *Actinidia arguta* Fruits. Pol. J. Food Nutr. Sci..

[44] Kabaluk J.T., Kempler C., Toivonen P.M.A. (1997). *Actinidia arguta*—Characteristics Relevant to Commercial Production. Fruit Var. J..

[45] Baldwin E.A., Nisperos-Carriedo M.O., Baker R.A. (1995). Use of Edible Coatings to Preserve Quality of Lightly (and Slightly) Processed Products. Crit. Rev. Food Sci. Nutr..

[46] Fenech M., Amaya I., Valpuesta V., Botella M.A. (2019). Vitamin C Content in Fruits: Biosynthesis and Regulation. Front. Plant Sci..

[47] Wang C., García-Caparros P., Li Z., Chen F., Wang C., García-Caparros P., Li Z., Chen F. (2024). A Comprehensive Review on Plant Ascorbic Acid. Trop. Plants.

[48] Hasanuzzaman M., Bhuyan M.H.M.B., Anee T.I., Parvin K., Nahar K., Mahmud J.A., Fujita M. (2019). Regulation of Ascorbate-Glutathione Pathway in Mitigating Oxidative Damage in Plants under Abiotic Stress. Antioxidants.

[49] Yin X., Chen K., Cheng H., Chen X., Feng S., Song Y., Liang L. (2022). Chemical Stability of Ascorbic Acid Integrated into Commercial Products: A Review on Bioactivity and Delivery Technology. Antioxidants.

[50] You J., Chan Z. (2015). ROS Regulation During Abiotic Stress Responses in Crop Plants. Front. Plant Sci..

[51] Krupa T., Latocha P., Liwińska A. (2011). Changes of Physicochemical Quality, Phenolics and Vitamin C Content in Hardy Kiwifruit (*Actinidia arguta* and Its Hybrid) during Storage. Sci. Hortic..

[52] Zhang R., Guo X., Zhang Y., Tian C. (2020). Influence of Modified Atmosphere Treatment on Post-Harvest Reactive Oxygen Metabolism of Pomegranate Peels. Natural Prod. Res..

[53] Zhou Y., Liu J., Zhuo Q., Zhang K., Yan J., Tang B., Wei X., Lin L., Liu K. (2023). Exogenous Glutathione Maintains the Postharvest Quality of Mango Fruit by Modulating the Ascorbate-Glutathione Cycle. PeerJ.

[54] Stefaniak J., Przybył J.L., Latocha P., Łata B. (2020). Bioactive Compounds, Total Antioxidant Capacity and Yield of Kiwiberry Fruit under Different Nitrogen Regimes in Field Conditions. J. Sci. Food Agric..

[55] Piechowiak T., Grzelak-Błaszczyk K., Sójka M., Balawejder M. (2020). Changes in Phenolic Compounds Profile and Glutathione Status in Raspberry Fruit during Storage in Ozone-Enriched Atmosphere. Postharvest Biol. Technol..

[56] Yao M., Ge W., Zhou Q., Zhou X., Luo M., Zhao Y., Wei B., Ji S. (2021). Exogenous Glutathione Alleviates Chilling Injury in Postharvest Bell Pepper by Modulating the Ascorbate-Glutathione (AsA-GSH) Cycle. Food Chem..

[57] Wołosiak R., Drużyńska B., Derewiaka D., Piecyk M., Majewska E., Ciecierska M., Worobiej E., Pakosz P. (2022). Verification of the Conditions for Determination of Antioxidant Activity by ABTS and DPPH Assays—A Practical Approach. Molecules.

[58] Szpadzik E., Zaraś-Januszkiewicz E., Krupa T. (2021). Storage Quality Characteristic of Two Minikiwi Fruit (*Actinidia arguta* (Siebold & Zucc.) Planch. Ex Miq.) Cultivars: ‘Ananasnaya’ and ‘Bingo’—A New One Selected in Poland. Agronomy.

[59] Choi H.R., Baek M.W., Tilahun S., Jeong C.S. (2022). Long-Term Cold Storage Affects Metabolites, Antioxidant Activities, and Ripening and Stress-Related Genes of Kiwifruit Cultivars. Postharvest Biol. Technol..

[60] Wojdyło A., Oszmiański J., Teleszko M., Sokół-Łętowska A. (2013). Composition and Quantification of Major Polyphenolic Compounds, Antioxidant Activity and Colour Properties of Quince and Mixed Quince Jams. Int. J. Food Sci. Nutr..

[61] Dong X., He Y., Yuan C., Cheng X., Li G., Shan Y., Zhu X. (2022). Controlled Atmosphere Improves the Quality, Antioxidant Activity and Phenolic Content of Yellow Peach during the Shelf Life. Antioxidants.

[62] Tavarini S., Degl’Innocenti E., Remorini D., Massai R., Guidi L. (2008). Antioxidant Capacity, Ascorbic Acid, Total Phenols and Carotenoids Changes during Harvest and after Storage of Hayward Kiwifruit. Food Chem..

[63] Abd-Elbaset A.A.A., Soliman M.A.E., El-Sherpiny M.A., Baddour A.G., Ghazi D.A., Abdelgawad Z.A., Abdein M.A., Alzuaibr F.M., Alasmari A., Albogami A. (2000). AOAC International Official Methods of Analysis.

[64] (2000). Soki Owocowe i Warzywne—Oznaczanie Zawartości Substancji Rozpuszczalnych Metodą Refraktometryczną.

[65] (2000). Soki Owocowe i Warzywne—Oznaczanie Kwasowości Miareczkowej.

[66] Singleton V.L., Rossi J.A. (1965). Colorimetry of Total Phenolics with Phosphomolybdic-Phosphotungstic Acid Reagents. Am. J. Enol. Vitic..

[67] Re R., Pellegrini N., Proteggente A., Pannala A., Yang M., Rice-Evans C. (1999). Antioxidant Activity Applying an Improved ABTS Radical Cation Decolorization Assay. Free. Radic. Biol. Med..

[68] Apak R., Güçlü K., Özyürek M., Çelik S.E. (2008). Mechanism of Antioxidant Capacity Assays and the CUPRAC (Cupric Ion Reducing Antioxidant Capacity) Assay. Microchim. Acta.

[69] Bountagkidou O.G., Ordoudi S.A., Tsimidou M.Z. (2010). Structure–Antioxidant Activity Relationship Study of Natural Hydroxybenzaldehydes Using *in Vitro* Assays. Food Res. Int..

[70] Di Majo D., La Neve L., La Guardia M., Casuccio A., Giammanco M. (2011). The Influence of Two Different pH Levels on the Antioxidant Properties of Flavonols, Flavan-3-Ols, Phenolic Acids and Aldehyde Compounds Analysed in Synthetic Wine and in a Phosphate Buffer. J. Food Comp. Anal..

[71] Lai L.-S., Chou S.-T., Chao W.-W. (2001). Studies on the Antioxidative Activities of Hsian-Tsao (*Mesona Procumbens* Hemsl) Leaf Gum. J. Agric. Food Chem..

